# The corneal subbasal nerve plexus and thickness of the retinal layers in pediatric type 1 diabetes and matched controls

**DOI:** 10.1038/s41598-017-18284-z

**Published:** 2018-01-08

**Authors:** Aline Götze, Sophie von Keyserlingk, Sabine Peschel, Ulrike Jacoby, Corinna Schreiver, Bernd Köhler, Stephan Allgeier, Karsten Winter, Martin Röhlig, Anselm Jünemann, Rainer Guthoff, Oliver Stachs, Dagmar-C. Fischer

**Affiliations:** 1 Department of Ophthalmology, Rostock University Medical Center, Rostock, Germany; 2 Department of Pediatrics, Rostock University Medical Center, Rostock, Germany; 3 Institute for Applied Computer Science, Karlsruhe Institute of Technology (KIT), Karlsruhe, Germany; 4 Institute for Anatomy, University of Leipzig, Leipzig, Germany; 5 Institute of Computer Science, University of Rostock, Rostock, Germany; 6 Department of Ophthalmology, Medical Faculty, Heinrich-Heine University, Düsseldorf, Germany

## Abstract

Optical coherence tomography (OCT) of the retina and corneal confocal laser scanning microscopy (CLSM) of the subbasal nerve plexus (SBP) are noninvasive techniques for quantification of the ocular neurodegenerative changes in individuals with type 1 diabetes mellitus (T1DM). In adult T1DM patients these changes are hardly related to T1DM only. Instead, ageing and/or lifestyle associated comorbidities have to be considered as putative confounding variables. Therefore, we investigated pediatric T1DM patients (n = 28; 14.2 ± 2.51 y; duration of disease: 5.39 ± 4.16 y) without clinical signs of diabetic retina disease, neuropathy, vasculopathy or nephropathy and compared our findings with those obtained in healthy controls (n = 46; 14.8 ± 1.89 y). The SBP was characterized by the averaged length, thickness, and tortuosity of nerve fibers as well as the number of branching and connecting points. OCT was used to determine the total thickness of the retina (ALL) and the thickness of each retinal layer. Both methods revealed signs of early neurodegenerative changes, e.g. thinning of distinct retinal layers at the pericentral ring and shortening of corneal nerve fibers that are already present in pediatric T1DM patients. Standardization of instruments and algorithms are urgently required to enable uniform comparison between different groups and define normative values to introduce in the clinical setting.

## Introduction

Type 1 diabetes mellitus (T1DM) is one of the most common metabolic disorders in childhood and affects nearly 15 million children worldwide^1^. On the long run, children with T1DM experience an increased risk of complications and comorbidities, i.e. vascular, renal, neurological and ophthalmological diseases Since the onset of the disease in the vast majority of T1DM patients occurs during childhood, these patients are at risk to suffer from diabetes-related complications already at young adulthood^2^.

Contrasting with the rather high sensitivity of the retina towards metabolic changes, virtually all forms of retinopathies remain asymptomatic for a long time^3^. There is increasing evidence that retinal neurodegeneration precedes diabetic retinopathy (DR) with first signs of microaneurysms^3–7^.

Optical coherence tomography (OCT) was introduced as a powerful tool for imaging and quantitative analysis of the retina^8^. OCT provides a cross-sectional view of the retina that allows discrimination and characterization of individual layers within the retina. Very recently, OCT revealed distinct but significant thinning of retinal layers in adults as well as adolescents and young adults suffering from T1DM^3,9^.

Diabetes can impair corneal sensitivity and diabetic neuropathy may also influence the subbasal nerve plexus (SBP) of the cornea^10,11^. Furthermore, it is already known that the condition of the SBP worsens not only with duration of the disease but also when glycemic control is insufficient and during the progression of diabetic polyneuropathy (DPN)^12–14^. In fact, confocal laser scanning microscopy (CLSM) revealed significant changes of the SBP in diabetic patients regardless of concomitant DPN^7,14–19^. However, quantitative analysis of CLSM images is still a challenging task as it requires sophisticated software tools to describe the SBP in terms of nerve fiber length, density, connectivity and tortuosity^7,14,19–21^.

However, there are still many open questions regarding the noted changes at the level of the SBP and retinal layers in T1DM patients relative to controls, since the significance of the OCT and CLSM findings is limited^9^. Whereas a wide variety of comorbidities have to be considered as putative confounding variables in adult T1DM patients, this is less relevant in pediatric T1DM patients. Consequently, cross-sectional studies in children and adolescents can open the window and may contribute to our understanding of neurodegenerative eye diseases. We hypothesize that in otherwise healthy pediatric T1DM patients, signs of neurodegenerative disease are already detectable with OCT and/or CLSM. In the present study, we report the results of a cross-sectional study obtained by applying both techniques simultaneously in a large cohort of pediatric T1DM patients and healthy controls matched for sex and age.

## Results

A total of 28 patients (18 males) with a mean age of 14.2 ± 2.51 years and 46 healthy volunteers (18 males) with a mean age of 14.8 ± 1.89 years consented to participate and CLSM of the eye was performed. Out of these, a subgroup of 26 patients (16 males) and 30 controls (14 males) received an additional OCT. Anthropometric and clinical data are given in Table 1. While anthropometric characteristics and age were fairly comparable between patients and controls, office blood pressure (BP) was significantly higher in patients compared to controls. Insulin was administered via multiple daily insulin injections (MDI) to 16 (15) patients and via continuous subcutaneous insulin infusion (CSII) to 12 (11) patients undergoing CLSM and OCT, respectively. Categorization of patients according to therapy revealed no differences with respect to the (*i*) distribution of boys and girls, (*ii*) duration of disease, (*iii*) daily insulin dosage, and (*iv*) glycemic control in terms of actual and mean HbA_1c_ (Supplemental Table 1). In all participants, visus was 1.0 or better and corneal sensitivity was well preserved (data not shown). At the time of enrolment, patients had neither clinical signs of diabetic vasculopathy, nephropathy or neuropathy nor mentioned such symptoms during the interview.

**Table 1:** Anthropometric and clinical characteristics of patients and controls.

	CLSM		OCT and CLSM	
	Patients (10 f/18 m)	Controls (28 f/18 m)	Patients (10 f/16 m)	Controls (16 f/14 m)
Age [year]	14.2 ± 2.54	14.8 ± 1.89	14.5 ± 2.23	14.5 ± 1.98
Height [SDS]	0.06 ± 0.88	0.58 ± 1.20	0.15 ± 0.89	0.66 ± 1.20
Weight [SDS]	0.23 ± 0.93	0.46 ± 1.00	0.31 ± 0.97	0.59 ± 0.94
BMI [SDS]	0.25 ± 0.92	0.26 ± 0.84	0.30 ± 0.96	0.37 ± 0.80
BP_sys_ [SDS]	1.94 ± 1.17^*^	1.27 ± 1.16^*^	1.95 ± 1.20	1.39 ± 1.18
BP_dias_ [SDS]	0.89 ± 0.74^*^	0.36 ± 0.88^*^	0.90 ± 0.76	0.50 ± 0.97
Duration of disease [year]	4.23 (1.2–15.5)		4.44 (1.2–15.5)	
Mean daily insulin dosage [IU/kg]	0.38 ± 0.15		0.39 ± 0.14	
Actual HbA_1c_ [%]	8.73 ± 2.00		8.96 ± 1.86	
Mean HbA_1c_ [%]	8.64 ± 1.53		8.73 ± 1.51	

Results are given as mean ± standard deviation and as median (min, max) for all participants undergoing CLSM and for the subgroups undergoing OCT and CLSM. *Significant difference between patients and controls.

### OCT and analysis of the retina

**Figure 1:**
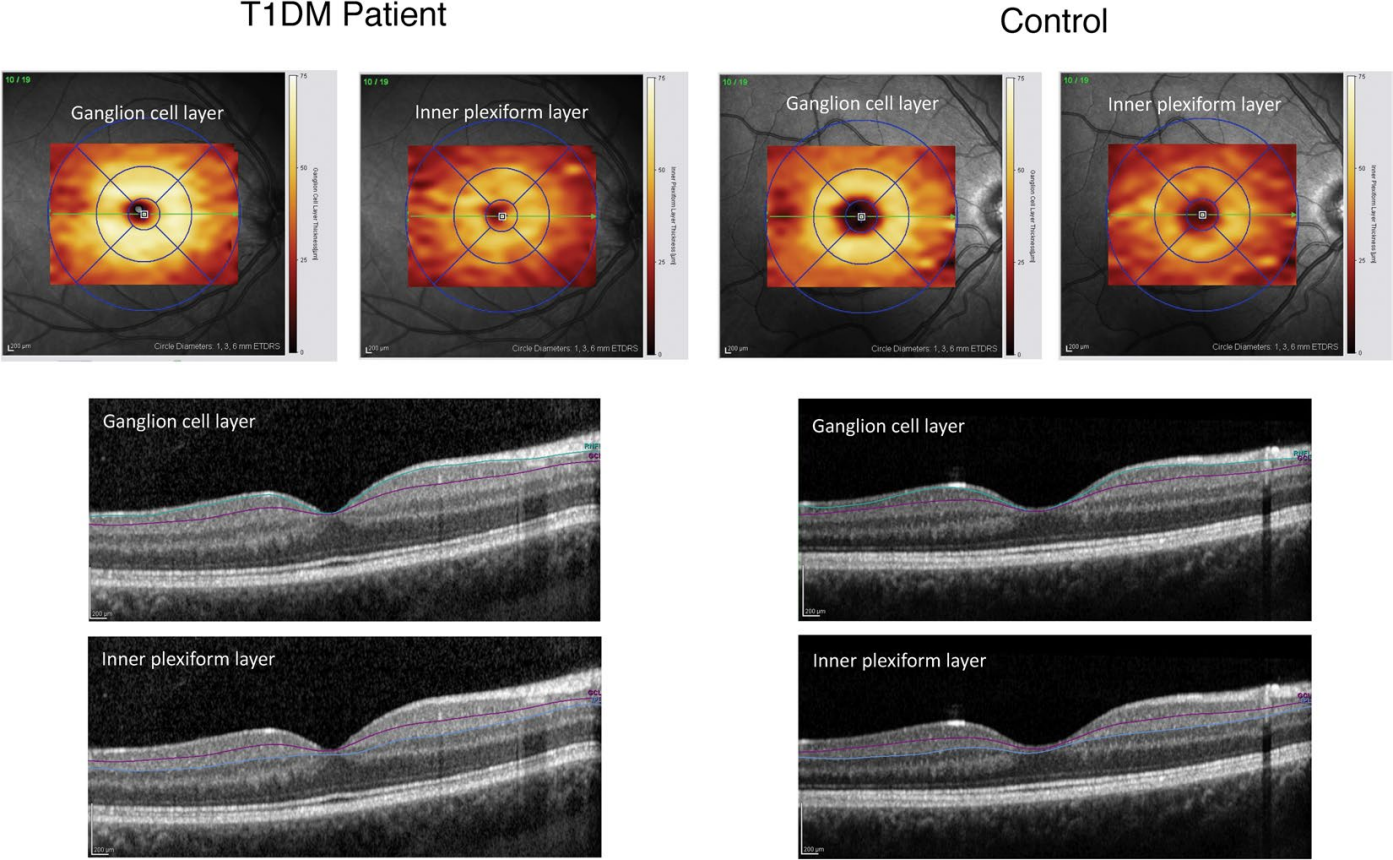
Representative images from a T1DM patient (left) and control (right). Automatically generated thickness maps and segmented boundaries of ganglion cell layer and the inner plexiform layer were presented.

**Figure 2:**
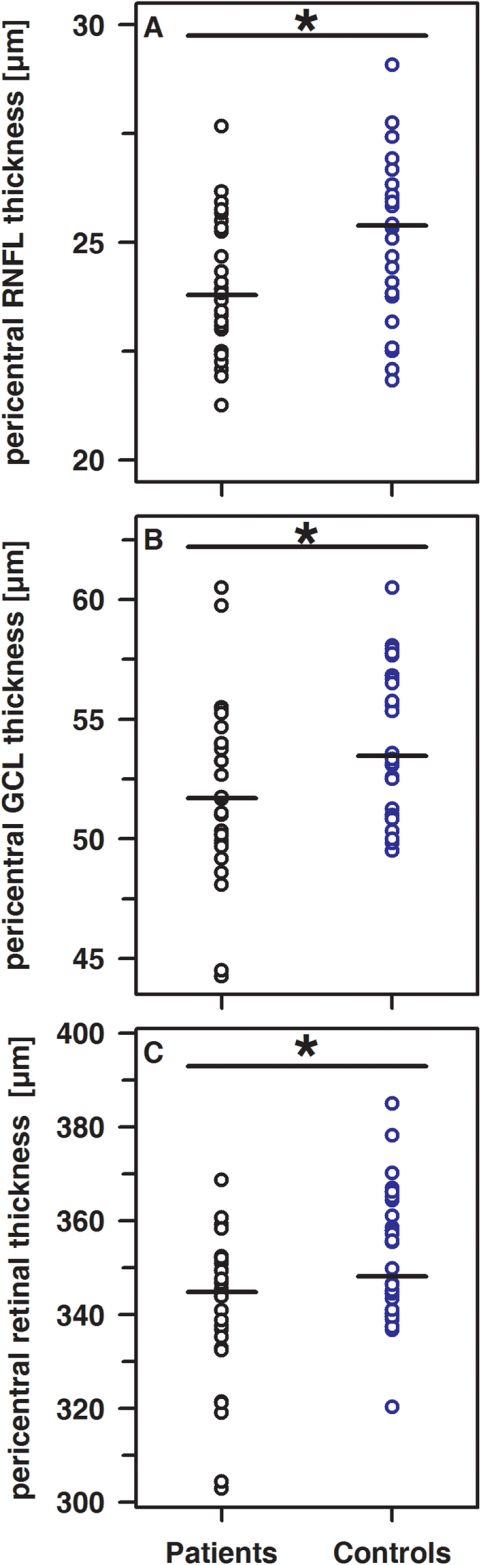
Significant differences (each p < 0.05) of the pericentral thickness of the RNFL (**A**), the GCL (**B**) and ALL (**C**) in patients (black circles) and controls (blue circles).

Typical images taken from the retina of a T1DM patient and a healthy control together with segmentation of the retinal layers by means of OCT are shown in Fig. 1. In healthy controls, the thickness of the retinal layers at all predefined sites of measurement turned out to be independent of age and sex. Therefore, these two variables were not considered for interpretation of the results. Total retinal thickness, as well as the thickness of individual retinal layers at the fovea and throughout the peripheral region, was quite similar in patients and controls (Supplemental Table 2). By contrast, within the pericentral ring, significant thinning of the retinal nerve fiber layer (RNFL), the ganglion cell layer (GCL), and total retinal thickness (ALL) was noted in T1DM patients compared to controls (Fig. 2). The thickness of the retinal layers was not associated with HbA_1c_ or duration of disease. Concerning the insulin regimen, patients on CSII revealed significantly thinner foveal GCL, inner plexiform layer (IPL), inner nuclear layer (INL), outer plexiform layer (OPL), and ALL compared to those on MDI (Table 2). Furthermore, in patients on CSII, foveal OPL and ALL was even significantly thinner compared to healthy controls. By contrast, in patients on MDI, none of these parameters was significantly different to healthy controls. However, OPL at the pericentral ring was significantly thinner in patients on MDI compared to those using CSII (Table 2).

**Table 2:** Thickness of the foveal retinal layers and of the pericentral OPL in patients on MDI and CSII therapy.

	CSII (5 f/6 m)	MDI (6 f/9 m)	p
Foveal thickness of the			
GCL [µm]	15.33 (10.00–21.67)	18.67 (14.00–42.67)	0.038
IPL [µm]	20.00 (17.67–26.33)	25.00 (20.33–38.33)	0.035
INL [µm]	17.67 (12.67–24.67)	20.33 (17.00–32.67)	0.021
OPL [µm]	25.67^*^ (16.33–32.33)	29.33 (21.33–36.33)	0.038
ALL [µm]	270.7^*^ (232.0–302.0)	290.0 (256.7–327.3)	0.024
pericentral thickness of the			
OPL [µm]	34.58 (30.08–40.50)	32.58 (28.42–35.08)	0.029

Data are given as median and range. ALL, total thickness of retina; GCL, ganglion cell layer; IPL, inner plexiform layer; INL, inner nuclear layer; OPL, outer plexiform layer. *p < 0.05 compared to controls.

Within the study population, the foveal thickness of RNFL, GCL, IPL and INL were strongly associated among themselves as well as with the total thickness of the retina (each R ≥ 0.74 and p < 0.001). Within the pericentral and peripheral area, the thickness of the GCL and IPL (each R ≥ 0.82 and p < 0.001) were strongly associated. The thickness of the outer nuclear layer (ONL), the OPL or the retinal pigment epithelium (RPE) turned out to be independent of the dimension of the inner retinal layers.

### CLSM and characteristics of the SBP

**Figure 3:**
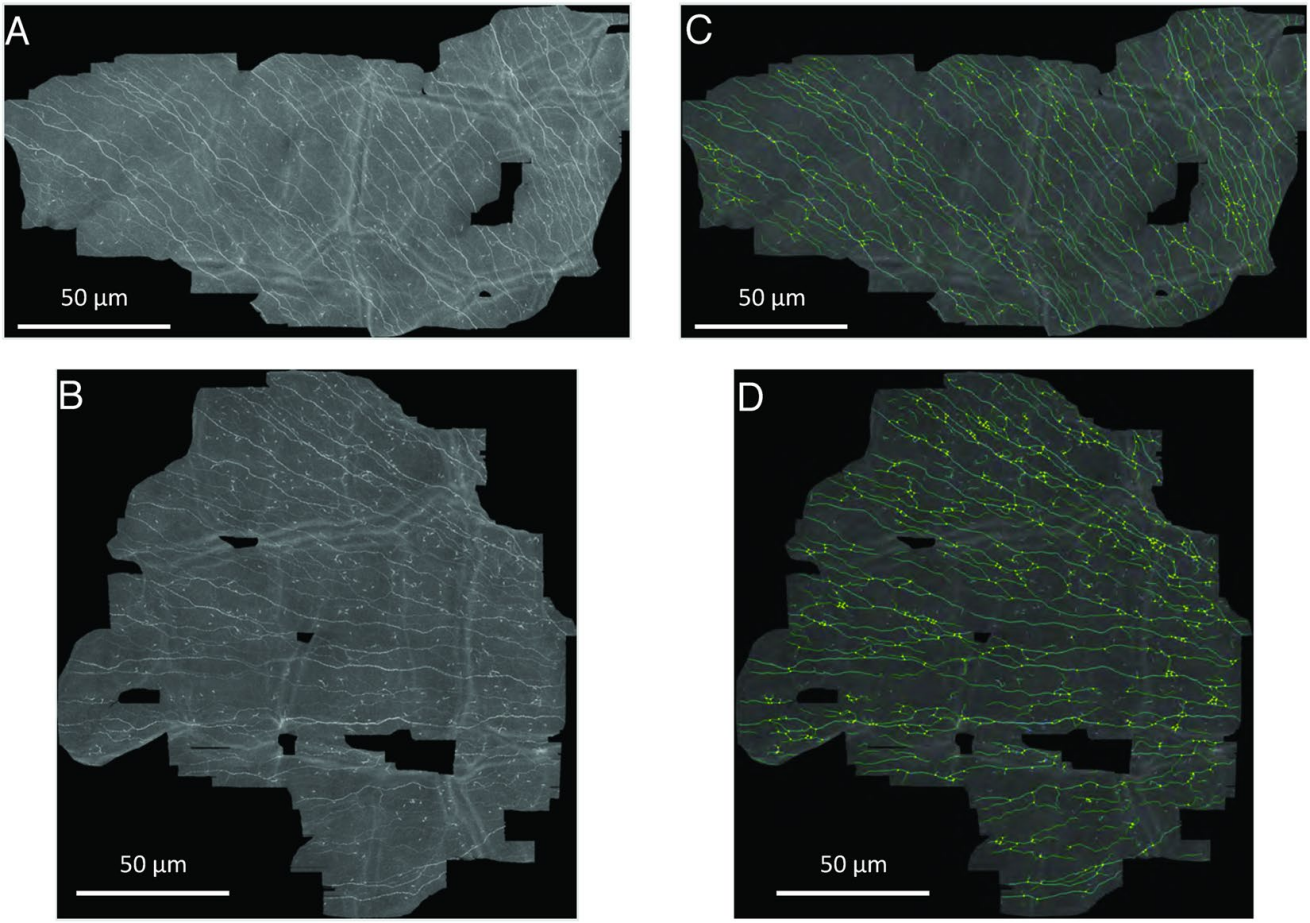
The typical mosaic image of the SBP of a patient (**A**), healthy control (**B**) and schematic representation of skeletonized images used for characterization of the SBP in patients (**C**) and healthy controls (**D**).

**Figure 4:**
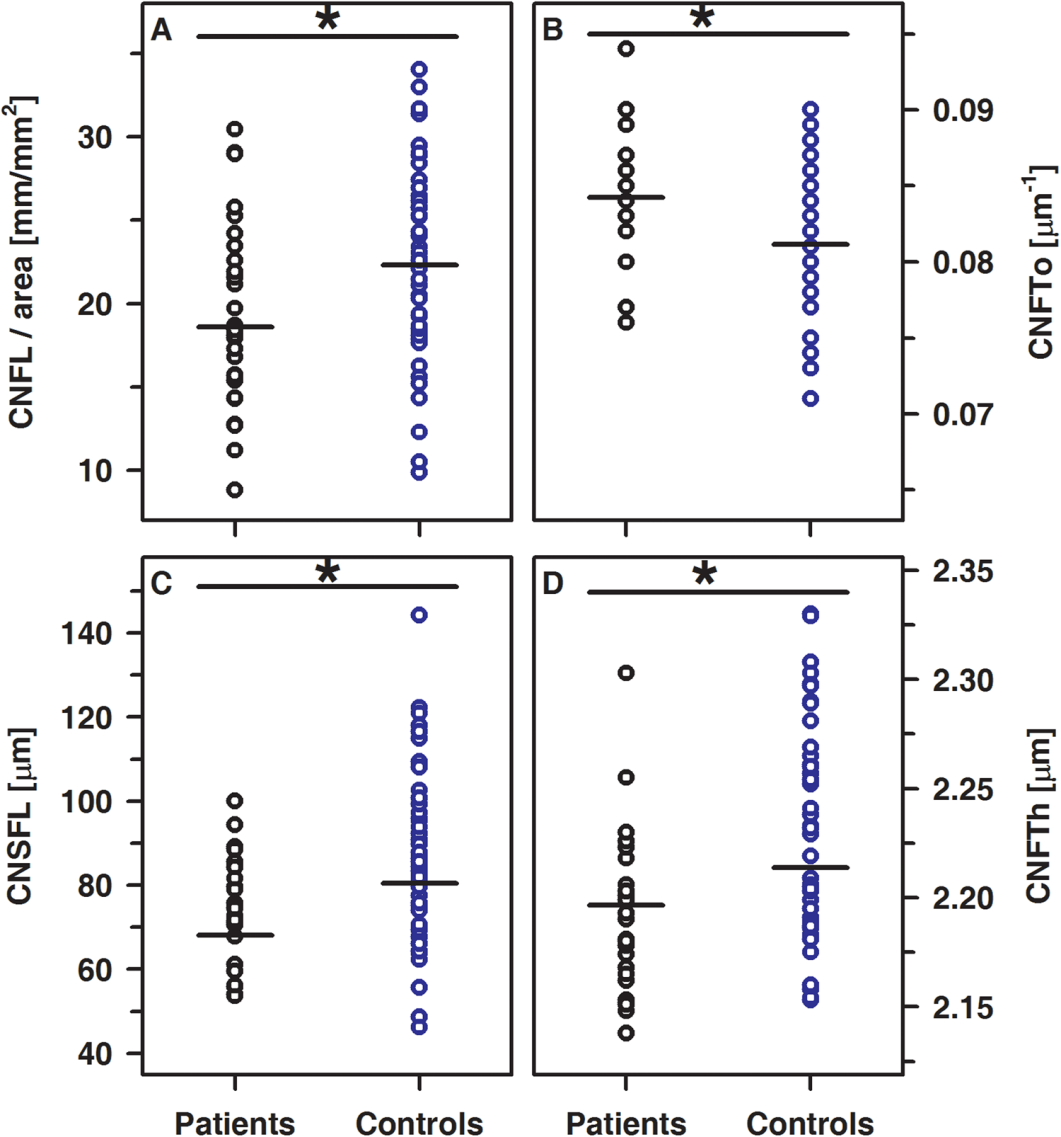
Significant differences between CNFL (**A**) p < 0.05), CNFTo (**B**) p < 0.01), CNSFL (**C**) p < 0.005) and CNFTh (**D**) p < 0.005) in patients (black circles) and controls (blue circles).

The SBP was analyzed in all participants and representative images were shown in Fig. 3. Corneal nerve fiber length (CNFL), corneal nerve single fiber length (CNSFL), and corneal nerve fiber thickness (CNFTh) were significantly lower and corneal nerve fiber tortuosity (CNFTo) is significantly higher in patients compared to healthy controls. Corneal nerve fiber density (CNFD), corneal nerve fiber branch density (CNBD), and the number of corneal nerve connecting points (CNCP) did not differ between groups (Fig. 4 and Table 3). In healthy controls, none of these parameters describing the SBP were related to age or sex. By contrast, in T1DM patients, CNFTo was significantly higher in females compared to males (0.086 µm^−1^ (range: 0.083–0.090 µm^−1^) vs. 0.083 µm^−1^ (range: 0.076–0.094 µm^−1^); p < 0.01). Furthermore, CNCP tended to be lower in female compared to male T1DM patients (60.75 mm^−1^ (range: 10.57–111.5 mm^−1^) vs. 87.99 mm^−1^ (range: 29.7–159.0 mm^−1^); p < 0.05). Although none of the parameters describing the SBP was associated with the HbA_1c_ or duration of disease, the difference might, at least partially, reflect that duration of disease was slightly longer in female compared to the male patients (5.19 years vs. 2.79 years; p = 0.10). The parameters describing the SBP were similar in patients on MDI and CSII (data not shown). In healthy controls, CNFL was strongly associated with CNFD, CNBD (each R = 0.95 and p < 0.001) and CNCP (R = 0.65, p < 0.001), while CNFTh and CNFTo were virtually independent of either one of these parameters used to describe the SBP. By contrast, in T1DM patients, CNFTh is related to CNFL (R = 0.411, p < 0.05) and CNCP (R = 0.544; p < 0.005). Furthermore, CNFL decreases reciprocally to tortuosity (R = −0.61; p ≤ 0.001).

**Table 3:** Results of the CLSM in pediatric T1DM patients and healthy controls.

	Patients (10 f/18 m)	Controls (28 f/18 m)	p
Results for			
CNFD [mm^−2^]	249 (84.0–503)	252 (126–641)	0.832
CNBD [mm^−2^]	135 (27.9–302)	123 (52.4–397)	0.798
CNCP [mm^−2^]	73.9 (10.6–159)	77.3 (22.6–153)	0.196

CNFD, corneal nerve fiber density; CNBD, corneal nerve fiber branch density; CNCP, corneal nerve connecting points.

### Correlation analysis of OCT and CLSM data

**Figure 5:**
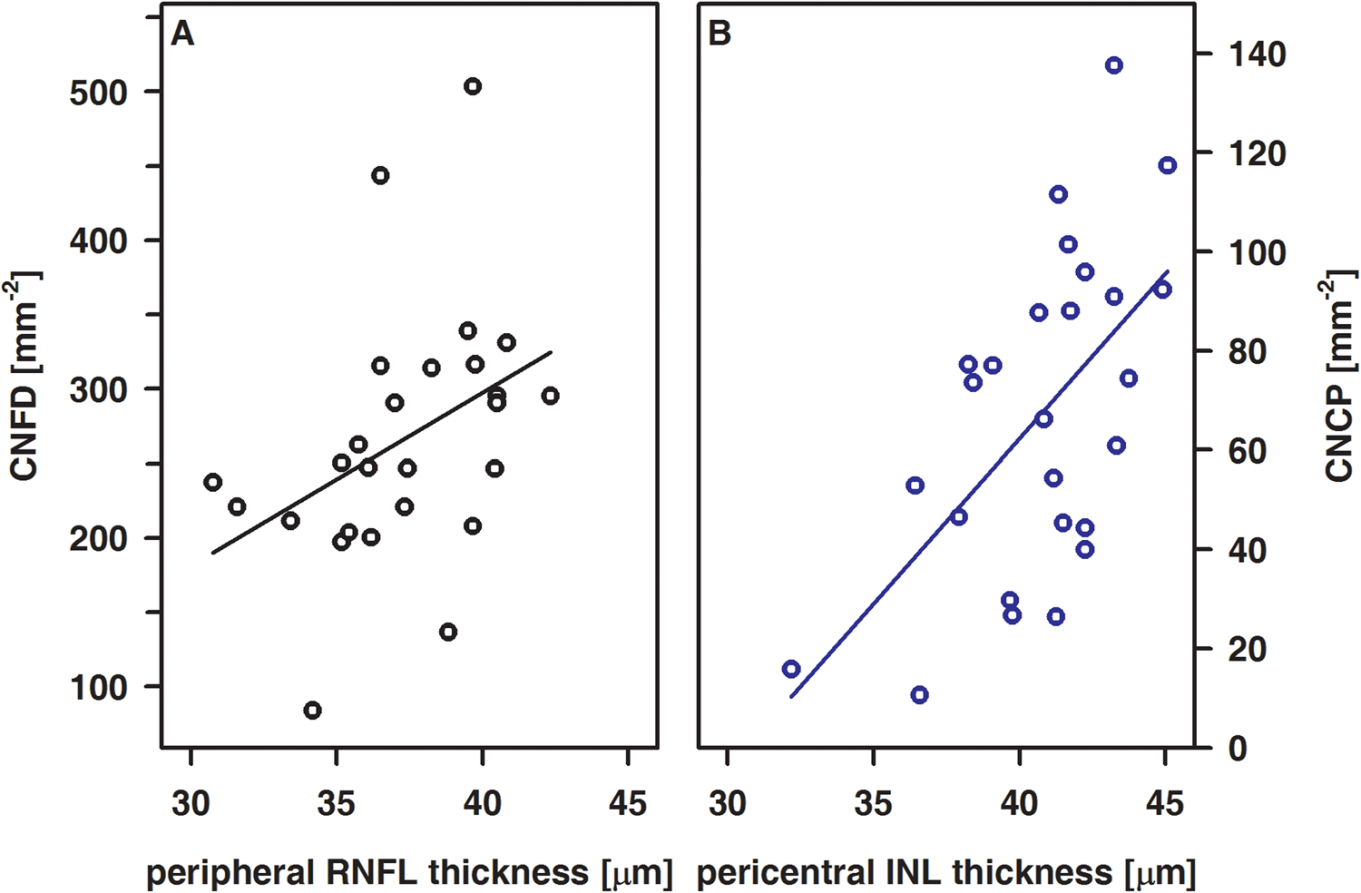
Correlations between CNFD and the peripheral RNFL thickness (**A**) and between CNCP and the pericentral INL thickness (**B**) in T1DM patients. A, R = 0.52, p < 0.01; B, R = 0.54, p < 0.01.

**Figure 6:**
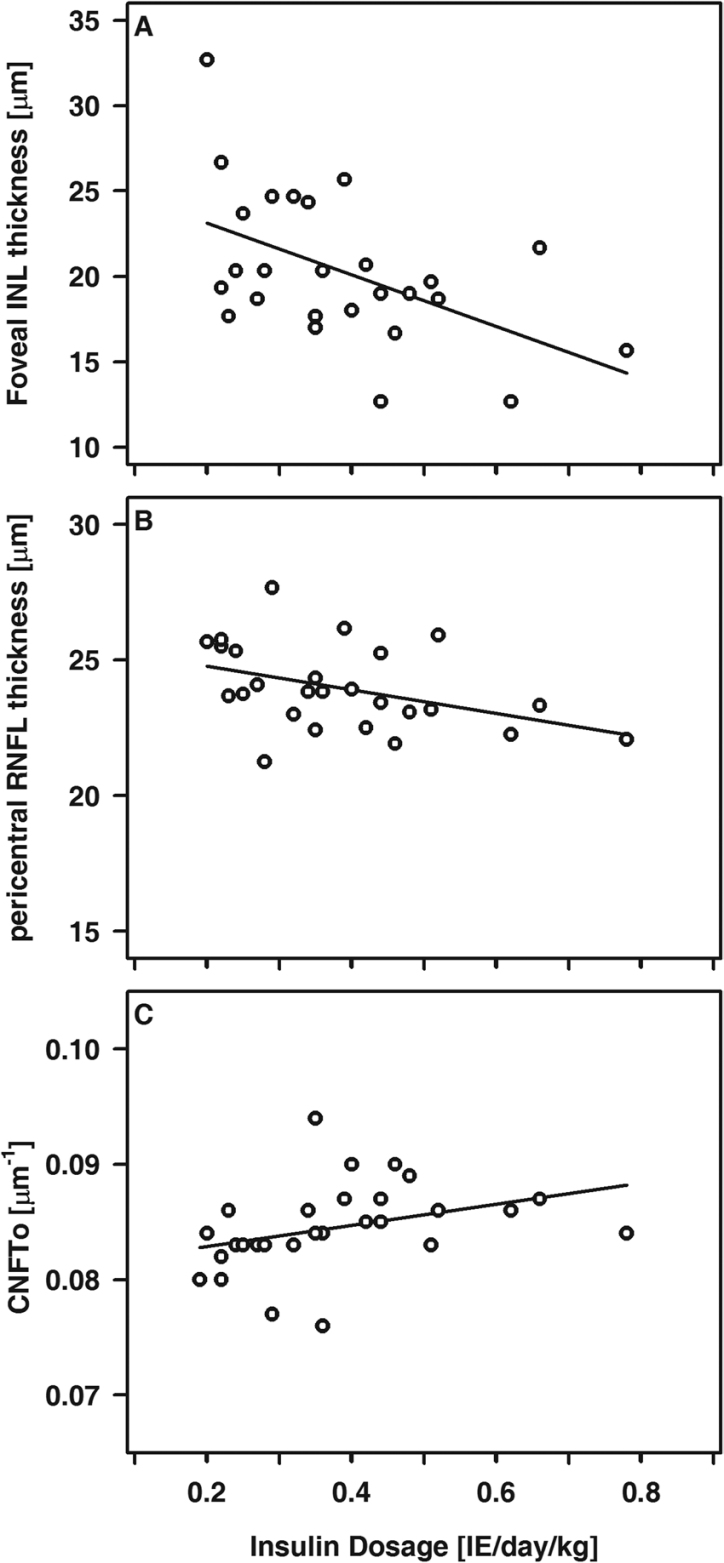
Foveal INL thickness (**A**), pericentral RNFL thickness (**B**) and CNFTo (**C**) relative to the basal insulin dosage in T1DM patients. A, R = −0.48, p < 0.05; B, R = −0.43, p < 0.05; C, R = 0.55, p < 0.005.

Within healthy controls, data obtained by OCT and CLSM were not associated at all. By contrast, in T1DM patients, the thickness of the RNFL at the peripheral area as well as the thickness of the INL at the pericentral area turned out to be significantly associated with CNFD and CNCP, respectively (Fig. 5). Although neuronal changes at the level of the retina and cornea turned out to be unrelated to glycemic control and duration of disease, CNFTo increases whereas foveal INL and pericentral RNFL thickness each decreases with insulin basal dosage rise (Fig. 6).

## Discussion

Within this cross-sectional study, we sequentially applied OCT and CLSM to pediatric T1DM patients and healthy age-matched controls. Besides separate interpretation of OCT and CLSM data relative to the underlying disease, our approach enabled us to analyze the association of both in healthy and diseased children. This is especially interesting, as early signs of DR or DPN are rarely seen in pediatric T1DM patients with current pediatric daily care.

Within our patient cohort, OCT revealed significant thinning of the RNFL, the GCL, and ALL at the pericentral ring. These findings were not related to HbA_1c_ or duration of disease. Our results correspond with very recent reports and support the current notion that retinal neurodegeneration occurs early and prior to vascular retinopathy^3–5,22–26^. Neuronal apoptosis and the loss of ganglion cell bodies are considered to be mainly responsible for the thinning of the inner retinal layers^5,6,26^. In line with this, our data also point to the higher vulnerability of the RNFL and GCL relative to the other retinal layers, especially in the absence of any signs of diabetic vascular retinopathy. Interestingly, patients on CSII therapy presented with significantly thinner foveal retinal layers than those using MDI therapy despite comparable daily basal insulin dosages, glycemic control and disease duration. Whether this finding is due to sampling bias, the incidence of diabetic ketoacidosis or related to the insulin preparation, i.e. pharmacological properties and half-life of insulin applied as bolus (MDI) or continuously (CSII), remains to be elucidated. In streptozotocin-induced diabetic rats, insulin reduces retinal apoptosis thereby preserving morphology of the tissue^6^. Although CSII compared to MDI has been associated with better glycemic control, we obtained evidence that retinal thinning at the fovea is more pronounced in patients on CSII, whereas the pericentral OPL was apparently more affected in patients on MDI. Additional studies are required to verify these findings.

Within our patient cohort, we noted significant inverse associations between basal insulin dosage and either the thickness of the pericentral RNFL or the thickness of the foveal INL. Apart from effects related to the insulin regimen, glycemic variability is more pronounced in pediatric T1DM patients on MDI compared to CSII^27^. Hyperglycemic episodes are capable to activate alternative pathways finally leading to microvascular complications^27,28^. However, retinal neurodegeneration is an early event and most likely precedes microvascular damage, i.e. apoptosis of retinal neural cells impairs the blood-retinal barrier with subsequent development of microangiopathy and finally thickening of the retinal layer^29–32^. Longitudinal studies are required to investigate the time course of retinal changes relative to the progression of the disease and the degree of glycemic variability in detail. Furthermore, data from morphological and functional investigations are required for better interpretation of the clinical relevance of morphological changes.

Similar to OCT, CLSM revealed significant neuronal changes in our T1DM patients. Besides a reduction of CNFL, CNSFL, and CNFTh, an increased CNFTo was noted and CNFTo increases as either CNFL or CNCP decrease. These findings are in agreement with those seen in adult DM patients and point to neurodegenerative changes early during the time course of the disease^7,9,12,14,15,18,19,33^. To the best of our knowledge, the SBP has been investigated thus far only in a very small group of pediatric DM patients^34^. Although the anthropometric and clinical characteristics regarding the duration of disease and glycemic control of pediatric patients enrolled in either study are fairly comparable, the results are not. Most likely, these differences are due to different experimental settings, the local variability of the SBP, the number and localization of image stacks taken per eye and subsequent processing of the data^20,21,35–37^.

The strong correlation between neurodegenerative changes at the level of retina and cornea, the absence of any clinically relevant signs of DR or DPN together with the missing association between neurodegenerative changes and duration of disease or glycemic control point to a rather high vulnerability and maladaptive response of these structures to diabetes-related metabolic changes^29^. Recently, we demonstrated an impaired endothelial vasodilation secondary to local heating in pediatric T1DM patients by means of laser Doppler fluximetry for assessment of skin microcirculation^38^. This effect was due to an impaired axon reflex mediating neurogenic vasodilation^38^. Of note, this was seen in pediatric T1DM patients without any clinical signs of DR or DPN^38^.

Although we obtained evidence that subtle neurodegenerative changes are already detectable in pediatric T1DM patients, our study has limitations. Unfortunately, documentation of diabtic ketoacidosis is not part of the routine diagnostic pipeline in our outpatient clinic and OCT became available for this study only after the start of the subject enrolment. Even though we requested all the subjects (that were already examined with CLSM) to undergo an additional OCT examination, 2 of the patients and 14 controls refused to participate. Due to this, we could investigate a rather small number of patients and describe morphological changes, only. Comorbidities are usually less relevant in pediatric patients and clinical signs of DR, DPN or vasculopathy were not detectable at all. However, to clearly link neurodegenerative changes to the time course of the disease, carefully designed longitudinal studies along with a concomitant assessment of morphological and functional changes relative to long-term glycemic control and the therapeutic regimen are required.

Symptomatic DR or DPN are rarely seen in children with T1DM and daily care clinical techniques are not sensitive enough to detect neuropathies at an early stage when improvement of therapy has the potential to prevent progression or even to promote nerve regeneration^34,39–41^. In this regard, our data indicate that OCT and CLSM both are valuable tools to detect early neurodegenerative changes. However, standardization of instruments, algorithms and data processing is an immediate prerequisite to enable data comparison between different groups. Defining the normative values of various retinal and SBP parameters may further assist in the secure transition of these methodologies into the clinical setting.

## Material and Methods

### Study design

The study received appropriate ethics committee approval from the institutional review board (Rostock University Medical Centre Ethics committee) in accordance with the Declaration of Helsinki. All examinations were done in accordance with the relevant guidelines. Subjects and/or their parents gave written and informed consent for participating in the study. All participants received a voucher to appreciate for the additional time spent in the hospital environment.

Pediatric T1DM patients being treated at the university between October 2012 and December 2013 were invited to participate. Healthy age-matched controls were recruited from different schools in Rostock. *Inclusion criteria:* age 6–18 years, duration of disease at least 12 months, C-peptide below 0.3 nmol/l, stable therapeutic regimen with either multiple daily insulin injections (MDI) or continuous subcutaneous insulin infusions (CSII, pump therapy) for at least 3 months. *Exclusion criteria*: any case of febrile illness during the last three months, chronic auto-inflammatory disease (e.g. Crohn’s disease, rheumatoid arthritis), hepatitis, HIV, glucocorticoid treatment, liver-, renal-, or cardiac failure, hereditary dyslipidemia, neurological diseases including idiopathic small fibre neuropathy, clinical evidence of diabetic peripheral neuropathy, limited ability to cooperate, pregnancy, tumoral diseases or ophthalmological diseases especially myopia of more than 6 diopters, corneal and retinal disorders.

## Methods

All participants were seen in our outpatient clinic. Demographic and clinical data were gathered by interview and chart review (i.e. duration of disease, mode of therapy, mean daily insulin dosages, mean HbA_1c_ during the last year). A trained physician measured weight and height using electronic scales and a fixed stadiometer. Office blood pressure (BP) was measured according to the updated Task Force Report on high blood pressure by using an oscillometric device (Dinamap 1846SX; Critikon, Tampa, USA). Calculations of individual age- and sex-related standard deviation scores (SD scores) for height, weight, BMI and BP were done as previously described^42,43^.

### Laboratory and clinical data

In patients, the actual HbA_1c_ expressed as a percentage of total hemoglobin and blood glucose levels were determined in the institute laboratory. The mean HbA_1c_ during the last 12 months and the actual mean insulin dosage per day and body weight were calculated.

### Ophthalmological status

All of the patients underwent a complete ophthalmologic examination, i.e. determination of visual acuity, intraocular pressure and slit-lamp examination in mydriatic fundoscopy. Patients and controls underwent OCT (Spectralis; Heidelberg Engineering GmbH, Heidelberg, Germany) for analysis of the retina, corneal esthesiometry and CLSM (HRTII + RCM; Heidelberg Engineering GmbH, Heidelberg, Germany) for analysis of the SBP. All ophthalmological tests were performed by the same experienced ophthalmologist (SP) for all study participants.

### Optical coherence tomography and analysis of the retina

Unilateral spectral-domain OCT was performed by the same experienced ophthalmologist (SP) for all study participants essentially as described previously^44^. All examinations were done in triplicate and the average of the results was used for subsequent analysis. This approach allowed mapping the macula thickness and for subsequent quantitative image analysis three circular segments centered at the fovea were separated automatically according to the ETDRS grid, i.e. a central, pericentral and a peripheral ring with outer diameters of 1 mm, 3 mm and 6 mm, respectively^45^. Furthermore, the pericentral and peripheral areas were subdivided into quadrants (temporal, superior, nasal and inferior). Within each subfield, the retinal thickness (ALL), the retinal nerve fiber layer (RNFL), the ganglion cell layer (GCL), the inner nuclear layer (INL), the inner plexiform layer (IPL), the outer plexiform layer (OPL), the outer nuclear layer (ONL), the retinal pigment epithelium (RPE) and the photoreceptor layer (PR) were discriminated. For each layer, the thickness was measured fully automatically at the localizations indicated using the algorithm provided by the manufacturer. Subsequently, data derived for each of the quadrants from the pericentral and peripheral ring were averaged. Thus, per layer three values reflecting the thickness at the fovea, the pericentral and peripheral area were obtained.

### Corneal esthesiometry

Corneal esthesiometry was carried out using the Cochet-Bonnet esthesiometer (Luneau Ophthalmology, France). The nylon monofilament had a diameter of 0.12 mm and a fully extended length of 60 mm. The central, superior, inferior, nasal, and temporal cornea was touched once on each eye, beginning at a filament length of 60 mm. If a positive answer was not detected the filament length was shortened in steps of 5 mm each time and the procedure was repeated until there was a positive response. Corneal sensation was calculated as the mean obtained from the five corneal areas on each eye.

### *In vivo* confocal laser-scanning microscopy and analysis of the corneal subbasal nerve plexus

For unilateral *in vivo* examination of the cornea, the Heidelberg Retina Tomograph (HRT II) in combination with the Rostock Cornea Module (RCM) was used essentially as described previously^46,47^. Both eyes were anesthetized (Proparacaine 0.5% eye drops; Ursapharm, Saarbrücken, Germany) and covered with Vidisic gel (Bausch & Lomb/Dr. Mann Pharma, Berlin/Germany; refractive index 1.35). To prevent eye movements, the patients were asked to fixate one spot with the unexamined eye.

Imaging in the central region (at or close to the corneal apex and more than 0.5 mm apart from the inferior whorl) was performed using a dedicated scan modality at the level of basal cells, SBP, Bowman’s membrane and anterior stroma described earlier^47^. At least three scans per region and patient were recorded. The total duration of *in vivo* CLSM was about 15 minutes per patient. Subsequently, the SBP layer was detected automatically in each recorded depth scan and a mosaic image of the SBP was generated and submitted to quantitative image analysis^21,47,48^. The following SBP parameters were determined: corneal nerve fiber length (CNFL), defined as the total length of all nerve fibers per unit area (mm/mm^2^); corneal nerve fiber density (CNFD), defined as the number of nerve fibers per unit area (n/mm^2^); corneal nerve branch density (CNBD), defined as the number of branching points per unit area (n/mm^2^); average weighted corneal nerve fiber tortuosity (CNFTo), reflecting variability of nerve fiber directions and defined as absolute nerve fiber curvature/nerve fiber length (per μm); corneal nerve connection points (CNCP), defined as the number of nerve fibers crossing the area boundary (connections/mm^2^); average corneal nerve single-fiber length (CNSFL), defined as the average length of nerve fibers (μm); and average weighted corneal nerve fiber thickness (CNFTh), measured as mean thickness perpendicular to the nerve fiber course (μm)^21,47,49^.

### Statistical analysis

For statistical analysis, the SPSS Software package, version 22.0 (SPSS GmbH, Munich, Germany), was used. Normal distribution was evaluated by the Kolmogorov-Smirnov test and comparison between groups was done using Student’s t-test or Mann-Whitney U test, if appropriate. For computation of correlations, Spearman’s rho test was used. All p-values are two-sided and a p-value below 0.05 was considered significant. Data are given as mean ± standard deviation (sd) or median and range, where appropriate.

### Data availability statement

The datasets generated during and/or analyzed during the current study are available from the corresponding author on reasonable request.

## Electronic supplementary material

Supplementary Tables
