# Supplementary material for: The corneal subbasal nerve plexus and thickness of the retinal layers in pediatric type 1 diabetes and matched controls
Source: Sci Rep. 2018 Jan 8;8:14. doi: 10.1038/s41598-017-18284-z (PMC5758564; doi:10.1038/s41598-017-18284-z)
Supplement: Supplementary file 1 — Supplementary Tables [file 41598_2017_18284_MOESM1_ESM.pdf]

The corneal subbasal nerve plexus and thickness of the retinal layers in pediatric type 1 diabetes and matched controls

Aline Götze[1$], Sophie von Keyserlingk[2$], Sabine Peschel[1], Ulrike Jacoby[2], Corinna Schreiver[2], Bernd Köhler[3], Stephan Allgeier[3], Karsten Winter[4], Martin Röhlig[5], Anselm Jünemann[1], Rainer Guthoff[6], Oliver Stachs[1#], and Dagmar-C. Fischer[2#*]

[$, #] these authors contributed equally to this work

[1]Department of Ophthalmology, Rostock University Medical Center, Rostock, Germany; [2]Department of Pediatrics, Rostock University Medical Center, Rostock, Germany;
[3]Institute for Applied Computer Science, Karlsruhe Institute of Technology (KIT), Karlsruhe, Germany;
[4]Institute for Anatomy, University of Leipzig, Leipzig, Germany;
[5]Institute of Computer Science, University of Rostock, Rostock, Germany
[6]Department of Ophthalmology, Medical Faculty, Heinrich-Heine University, Düsseldorf, Germany

Financial support: This work has been supported by the German Research Foundation (Project VIES).

Conflict of Interest: No conflicting relationship exists for any author.

This article contains additional online-only material. The following should appear online-only: Tables S1 to S2

*Corresponding author / Address for reprints:

Dagmar-C. Fischer, Department of Pediatrics, Rostock University Medical Center

Ernst-Heydemann-Str. 8, 18057 Rostock, Germany

Tel:/Fax: +49 - 381 - 4 94 7041 / 70 44

e-Mail: dagmar-christiane.fischer@med.uni-rostock.de

Supplement:

Table 1: Anthropometric and clinical characteristics of patients with respect to the mode of insulin therapy.

Results are given as mean ± standard deviation and median (min, max) for patients using MDII and CSI, respectively.

Table 2: Results of the OCT in pediatric T1DM patients and healthy controls.

Total retinal thickness as well as the thickness per retinal layer at the fovea together with the averaged thickness of the pericentral and peripheral areas are given as median and range.

	CSII (5f / 7m)	MDI (6f / 10m)	p
Age [year]	13.2 (8.6 - 18.4)	15.0 (10.5 - 17.8)	0.302
Absolute height [cm]	164 (128 - 184)	165 (139 - 184)	0.982
Standardized height [SDS]	0.45 (-0.83 - 2.16)	0.20 (-1.59 - 0.96)	0.037
Absolute weight [kg]	53.8 (28.3 - 79.8)	55.7 (33.2 - 83.0)	0.664
Standardized weight [SDS]	0.27 (-0.94 - 2.00)	0.07 (-1.38 - 2.27)	0.397
Absolute BMI [kg/m^2]	19.7 (15.7 - 27.9)	20.9 (17.2 - 28.4)	0.423
Standardized BMI [SDS]	0.00 (-1.65 - 1.94)	0.30 (-1.63 - 2.07)	0.599
Absolute BP_{sys} [mmHg]	137 (105 - 148)	129 (113 - 166)	0.581
Standardized BP_{sys} [SDS]	2.40 (-0.49 - 3.56)	1.59 (0.58 - 4.44)	0.548
Absolute BP_{dias} [mmHg]	77 (64 - 94)	71 (57 - 86)	0.083
Standardized BP_{dias} [SDS]	1.21 (0.05 - 2.49)	0.70 (-0.87 - 1.76)	0.075
Duration of disease [years]	5.96 (1.2 - 13.1)	2.75 (1.2 - 15.5)	0.121
Mean daily insulin dosage [IU/kg]	0.30 (0.19 - 0.78)	0.37 (0.20 - 0.66)	0.324
Actual HbA1c [%]	8.45 (6.9 - 9.9)	9.2 (4.6 - 14.8)	0.568
Mean HbA1c [%]	8.1 (6.8 - 11.3)	8.5 (6.7 - 12.7)	1

Götze, von Keyserlingk et al, Supplemental Table 1

	Thickness [μm]	Patients	Controls	p
ALL	fovea	276.2 (232.0 - 327.3)	286.0 (244.0 - 319.0)	0.275
	pericentral	344.8 (302.9 - 368.7)	348.2 (320.3 - 385.0)	0.045
	peripheral	308.2 (283.2 - 336.8)	311.1 (282.6 - 334.9)	0.189
RNFL	fovea	15.67 (11.00 - 23.67)	15.33 (10.67 - 18.33)	0.786
	pericentral	23.79 (21.25 - 27.67)	25.38 (21.83 - 29.08)	0.022
	peripheral	37.17 (30.75 - 42.33)	39.42 (32.92 - 44.67)	0.066
GCL	fovea	17.83 (10.00 - 42.67)	18.33 (11.00 - 28.00)	0.506
	pericentral	51.78 (44.25 - 60.50)	53.46 (49.50 - 60.50)	0.039
	peripheral	37.63 (32.67 - 43.75)	38.04 (32.67 - 44.58)	0.582
IPL	fovea	22.83 (17.67 - 38.33)	23.00 (17.33 - 28.67)	0.961
	pericentral	42.13 (36.42 - 48.33)	43.46 (39.50 - 46.25)	0.137
	peripheral	30.63 (26.00 - 36.25)	31.58 (26.33 - 35.75)	0.571
INL	fovea	19.50 (12.67 - 32.67)	18.17 (12.33 - 30.00)	0.285
	pericentral	41.29 (32.17 - 45.08)	40.79 (35.92 - 46.58)	0.850
	peripheral	34.92 (31.50 - 39.58)	34.83 (31.75 - 40.33)	0.749
OPL	fovea	26.67 (16.33 - 36.33)	29.17 (18.67 - 38.00)	0.445
	pericentral	33.29 (28.42 - 40.50)	33.25 (27.08 - 41.25)	0.935
	peripheral	27.42 (24.42 - 31.50)	27.25 (25.08 - 31.17)	0.730
ONL	fovea	86.67 (66.67 - 104.0)	89.67 (47.33 - 108.3)	0.133
	pericentral	67.38 854.67 - 89.50)	71.29 (45.67 - 84.25)	0.215
	peripheral	59.79 (47.42 - 76.67)	61.17 (40.42 - 70.25)	0.341
RPE	fovea	17.33 (12.67 - 20.33)	17.67 (14.67 - 22.33)	0.273
	pericentral	14.38 (11.83 - 16.25)	14.79 (13.08 - 16.83)	0.349
	peripheral	13.17 (11.25 - 15.33)	13.38 (11.08 - 15.50)	0.967
PR	fovea	90.83 (80.67 - 96.33)	92.67 (83.00 - 98.67)	0.505
	pericentral	82.38 (71.83 - 86.17	83.08 (78.58 - 87.00)	0.139
	peripheral	79.75 (74.75 - 81.92)	80.21 (74.58 - 83.50)	0.285

Götze, von Keyserlingk et al, Supplemental Table 2
